# Feasibility and Acceptability of a Web-Based Treatment with Telephone Support for Postpartum Women With Anxiety: Randomized Controlled Trial

**DOI:** 10.2196/mental.9106

**Published:** 2018-04-20

**Authors:** Miriam T Ashford, Ellinor K Olander, Heather Rowe, Jane RW Fisher, Susan Ayers

**Affiliations:** ^1^ Centre for Maternal and Child Health Research School of Health Sciences City, University of London London United Kingdom; ^2^ Jean Hailes Research Unit School of Public Health and Preventive Medicine Monash University Melbourne Australia

**Keywords:** anxiety, mental health, postpartum period, treatment, Internet, randomized controlled trial

## Abstract

**Background:**

Postpartum anxiety can have adverse effects on the mother and child if left untreated. Time constraints and stigma are common barriers to postpartum treatment. Web-based treatments offer potential flexibility and anonymity. *What Am I Worried About* (WaWa) is a self-guided treatment based on cognitive-behavioral and mindfulness principles for women experiencing postpartum anxiety. WaWa was developed in Australia and consists of 9 modules with optional weekly telephone support. WaWa was adapted to a Web-based version for use in England (Internet-based What Am I Worried About, iWaWa).

**Objective:**

This study aimed to investigate the feasibility (engagement and usability) and acceptability (usefulness, satisfaction, and helpfulness) of iWaWa among English postpartum women with anxiety.

**Methods:**

Postpartum (<12 months) women with mild-to-severe anxiety were recruited anonymously via social media during an 8-week period. Participants were randomized to the iWaWa treatment (8 weeks) or wait-list control group. Treatment and study feasibility and acceptability were assessed after the treatment, and anxiety symptoms were assessed at baseline, 8 weeks postrandomization, and 12 weeks postrandomization (treatment group only) using Web-based questionnaires. Semistructured telephone interviews were carried out after the treatment period for a more in-depth exploration of treatment acceptability and feasibility.

**Results:**

A total of 89 eligible women were recruited through social media and randomized into the treatment (n=46) or wait-list control group (n=43). Women were predominantly Caucasian, well-educated, married, on maternity leave, first-time mothers and reported moderate levels of anxiety. Dropout rates were high, especially in the treatment group (treatment: 82%, 38/46; wait-list control: 51%, 22/43). A total of 26 women started iWaWa with only 2 women completing all 9 modules. Quantitative and qualitative data suggest iWaWa was experienced as generally useful and helpful. Participants enjoyed iWaWa’s accessibility, anonymity, and weekly reminders, as well as the introduction to the principles of cognitive-behavioral therapy (CBT) and mindfulness. However, iWaWa was also experienced as not user-friendly enough, too long, and not smartphone-friendly. Parts of the content were experienced as not always relevant and appropriate. Participants felt that iWaWa could be improved by having it in a smartphone app format and by making the content more concise and inclusive of different parenting styles.

**Conclusions:**

Despite interest in iWaWa, the results suggest that both the study and iWaWa were not feasible in the current format. However, this first trial provides useful evidence about treatment format and content preferences that can inform iWaWa’s future development, as well as research and development of Web-based postpartum anxiety treatments, in general, to optimize adherence.

**Trial Registration:**

ClinicalTrials.gov NCT02434406; https://clinicaltrials.gov/ct2/show/NCT02434406 (Archived by WebCite at http://www.webcitation.org/6xTq7Bwmd)

## Introduction

### Postpartum Anxiety

Anxiety disorders such as generalized anxiety disorder, obsessive-compulsive disorder, panic disorder, and phobias in the first year after birth (postpartum) are common with prevalence rates ranging between 9.9% and 20% [1-3]. Anxieties in the postpartum period are often life-stage specific, for example, worries about baby’s care and health and fear of criticism and inadequacy as a mother [4]. Postpartum anxiety disorders can either be a recurrence of a previous disorder or develop as a first episode. Symptom intensity and associated degree of impairment of these anxiety disorders can vary over the course of the postpartum period [5]. Despite available effective treatments [6-10], postpartum mental health problems often go undetected or untreated [11,12]. Low screening and diagnosis rates play a role, but some women with emotional difficulties postpartum are often more reluctant to disclose and seek help [13-15]. Possible reasons for this include being too busy to get around to seeking help and feeling too embarrassed or having no-one they felt comfortable talking to [14], as well as child care concerns [16].

The importance of having efficient and timely treatments is highlighted by the adverse effects of untreated mental health problems on the physical and psychological health of the mother, child, and family [17,18], as well as potential costs to society [11]. For example, it has been shown that maternal anxiety can affect infant bonding and feeding [19,20] and may negatively affect the child’s cognitive and social development [17]. Considering the importance of treatment and unique postpartum barriers to accessing treatment, providing convenient and anonymous treatment seems essential.

### Web-Based Self-Help Treatments

One approach of offering anonymous and convenient treatment is Web-based self-help treatments, which run on computers, tablets, or smartphones and allow individuals to work through written therapy material without or with minimal therapist or mental health professionals’ assistance. Many of today’s parents search for information and support on the Web [21]. In addition, postpartum women who feel isolated or restricted by their baby’s schedule experience Web-based resources as useful [22]. In a thematic analysis of motivators and barriers to a Web-based postpartum treatment, it was found that the offered flexibility and anonymity fitted women’s postpartum circumstances [23]. This suggests that Web-based treatments may be an appropriate alternative or supplement to conventional face-to-face therapy for postpartum women.

Systematic reviews and one meta-analysis focusing on the perinatal period suggest that Web-based treatments can help improve postpartum depressive symptoms [24-26], but so far none are specifically developed for postpartum anxiety [24]. A Web-based survey demonstrated that women with postpartum anxiety are interested in Web-based treatments [27], and a qualitative study of postpartum health care professionals (health visitors) in the United Kingdom reported a need for more treatment options for postpartum anxiety and that Web-based treatments could be useful to address this issue [28].

### The What Am I Worried About Treatment

On the basis of the identified interest and need, a self-help treatment for women experiencing moderate or severe symptoms of postpartum generalized anxiety disorder called *What Am I Worried About* (WaWa) developed in Australia [4] was transformed into a Web-based version called Internet-based WaWa (iWaWa). This study aimed to evaluate the feasibility and acceptability of iWaWa for women with postpartum anxiety problems in England. On the basis of the stage model of behavioral therapy research [29,30], the primary study objectives were to determine study feasibility by examining recruitment and attrition, examine iWaWa’s feasibility in terms of engagement and usability, and examine user’s acceptability of iWaWa in terms of usefulness, helpfulness, and satisfaction. The secondary objective was to examine potential changes in anxiety over the course of the treatment and compare with a wait-list control group.

## Methods

The study received ethical approval from the National Research Ethics Service, London—Dulwich Research Ethics Committee (ref: 15/LO/1827).

### Sample and Recruitment

#### Sample Size Calculation

Studies evaluating the feasibility of Web-based treatments for postpartum depression have recruited between 53 to 103 participants in total [31,32]. A power calculation indicated that 27 participants in each group would be required to achieve 95% power at a one-sided 5% significance level. In studies evaluating postpartum depression, Web-based treatments had attrition rates between 11.3% and 62.3%, with an average attrition of 34.2% [31,33,34]. About 18 more participants needed to be recruited to allow for 34.2% attrition. It was therefore aimed for a minimum of 36 participants per group (treatment vs wait-list control) (total=72).

#### Recruitment

Participants (n=89) were recruited over 8 weeks (March to May 2017) through Facebook, Twitter, and appropriate UK third-party parenthood websites, as well as through posters and flyers in two clinical settings in England (hospital and health visiting clinic). The development of the promotional material was informed by mothers participating in patient and public involvement meetings. Monetary compensation was only offered for taking part in the follow-up interviews.

#### Eligibility Criteria

Eligible participants had to have given birth in the last 12 months, be aged over 18 years, be living in England, be able to read and write English, have internet access, and have scored ≥5 on the Generalized Anxiety Disorder Scale (GAD-7) [35]. Women were excluded if they were receiving formal psychological treatment at the start of the study, reported self-harm or suicidal ideation, or had a stillbirth or the baby was seriously ill.

### The Web-Based Treatment (iWaWa)

#### Origin and Format

iWaWa is based on the WaWa self-help booklet for postpartum generalized anxiety disorder [4]. A licensing agreement with Monash University allowed researchers at the City, University of London to develop a Web-based version of WaWa for use in England in collaboration with the WaWa development team in Australia.

WaWa is based on cognitive-behavioral and mindfulness principles and consists of 3 sections: (1) Is this for me? (2) Practice, and (3) Understanding. In the first section, concepts such as generalized anxiety disorder, common worries during the perinatal period, and the cognitive-behavioral therapy (CBT) and mindfulness models are explained, and the program is outlined. The section on “Practice” consists of 7 worksheet modules that target life stage-specific anxieties and worries using guided activities. The last section provides background information about the biopsychosocial model of anxiety and a lay language description of CBT and mindfulness theories and practice. For more detailed information about the WaWa program, please refer to [4].

#### iWaWa Format

iWaWa, the Web-based WaWa version, was developed on and hosted by the Qualtrics Platform and a City University of London blog. For the Web-based format, the three main sections were divided into 9 modules (one “Is this for me?” module; seven “Practice” modules; and one “Understanding” module). A link to each module could be found on a password-protected blog page of the iWaWa study website. See Multimedia Appendix 1 for two images of iWaWa. Sessions were made up of multimedia presentations (text, images, and audio) and Web-based interactive material (eg, textboxes, self-assessment with sliders, hotspot graphics).

Participants were advised to start with the first module, but were free to access the remaining modules in any order and as many times as they wished. iWaWa users were also offered optional weekly email and or text-message reminders and weekly 30-min telephone support with each practice module. The iWaWa coach (MA) was a health psychology doctoral student with a master’s in clinical psychology. An adapted version of the WaWa Health Professional’s Guide was developed, which included checklists to record fidelity of program implementation and participant understanding and progress. No changes were made to iWaWa after the trial started.

### Study Design and Procedure

#### Design

**Figure 1:**
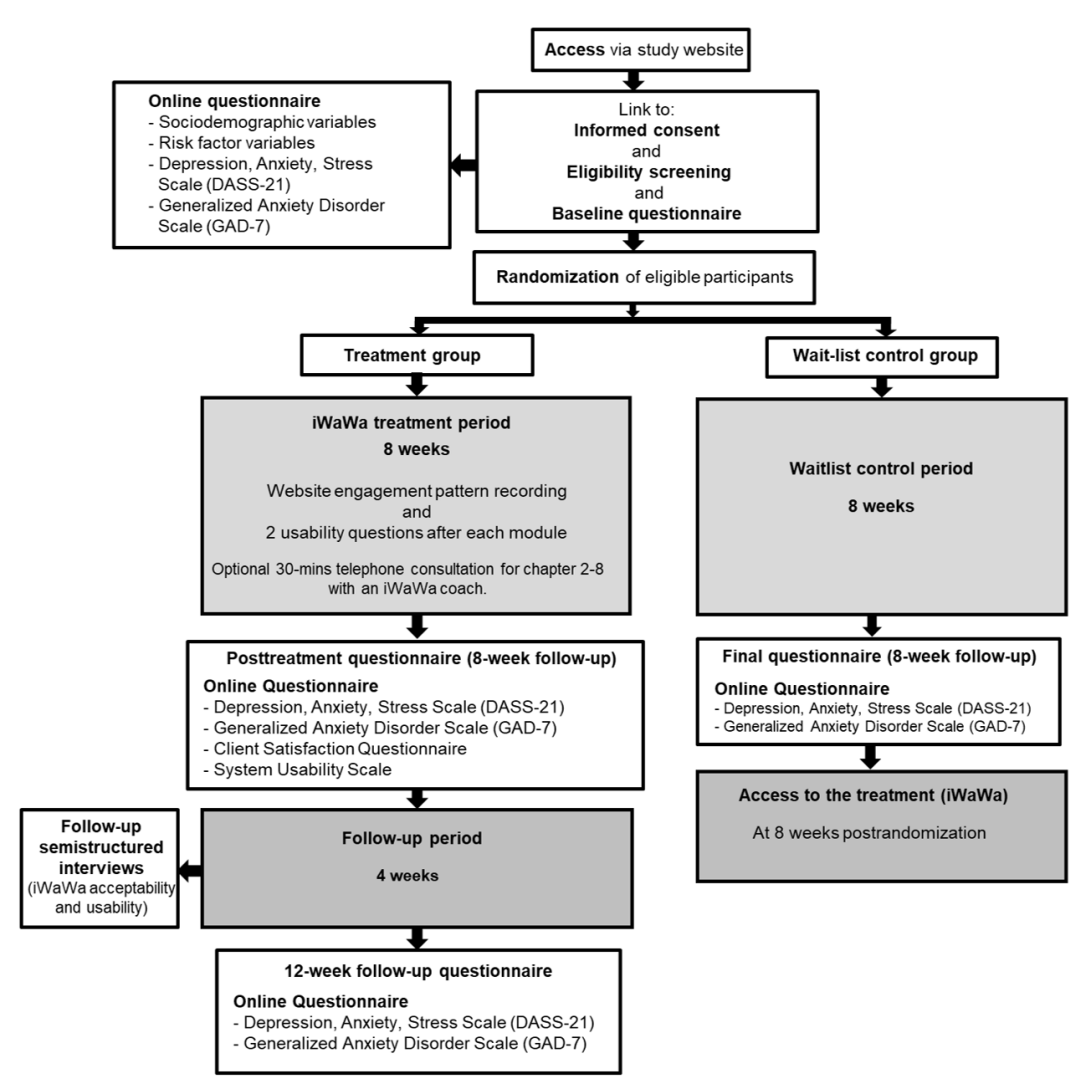
Study design and procedure flowchart. iWaWa: Internet-based What Am I Worried About.

Figure 1 illustrates the study design and procedures including data collection time-points and measures. A 2 (groups) by 3 (time-points) randomized controlled trial was carried out. Using a blocked randomization design (generated on the Web), the participants were randomly allocated to an iWaWa treatment group or a wait-list control group. Data were collected at baseline, throughout the treatment, 8 weeks postrandomization, and 12 weeks postrandomization utilizing both quantitative (Web-based questionnaires) and qualitative methods (optional iWaWa module comments and semistructured interviews).

#### Procedure

The study website contained a link to the Web-based questionnaire consisting of the electronic informed consent procedure, the eligibility questions, and the baseline assessment. Women who were not eligible were provided with links to websites of organizations dealing with postpartum or general mental health problems and advised to contact their general practitioner or health visitor if concerned about their mental health.

Participants were quasi-anonymous. Treatment allocation was revealed to eligible participants via email, and participants created a personal identifier for the iWaWa modules and Web-based assessments. The personal identifier was also used to detect participants signing up multiple times. Treatment group participants were immediately emailed the link and password to the iWaWa program with telephone support, and wait-list control group participants were offered access to iWaWa without telephone support at 8 weeks postrandomization. Treatment group participants received one reminder email to start treatment, and all participants received one reminder email for the 8-week and 12-week follow-up assessments. Participants and the researcher (MA) responsible for the study management and analysis were not blinded.

### Measures

#### Study Feasibility

For study feasibility, the following parameters were recorded: (1) recruitment rate and recruitment source, (2) eligibility and consent rates, (3) dropout attrition rates, and (4) completeness of data collection and assessment response rates.

#### Treatment Feasibility

##### Engagement

Module views (module opened) and completion (all pages of the module were viewed), engagement with interactive components, and the number and duration of iWaWa support calls were recorded. Nonusage attrition rates were calculated.

##### Usability

Upon completion of each module, participants were asked to rate the module’s clarity (“This module was clear and understandable.”) on a 7-point Likert scale (1=strongly disagree to 7=strongly agree). At the 8-week postrandomization assessment, the System Usability Scale (SUS) [36] was used to determine treatment usability. The SUS is a 10-item instrument rated on 5-point Likert scale (0=strongly disagree to 4=strongly agree). The SUS has been found to be a highly robust and versatile tool [36] and has previously been used in a study evaluating the feasibility of Web-based treatment for postpartum depression [31]. The SUS was adapted by replacing “the system product” with “iWaWa.” Scores were added together and multiplied by 2.5 to convert the original scores to 0-100. A score above a 68 is considered above average and below 68 is below average. Participants were also asked to rate how iWaWa fit into their daily routine and potential future usage on a 7-point Likert scale (1=strongly disagree to 7=strongly agree). Participants were further asked to state any technical issues.

#### Treatment Acceptability

##### Usefulness

At the end of each module, participants were asked to rate the module’s usefulness (“I found this module useful”) on a 7-point Likert scale (1=strongly disagree to 7=strongly agree). At the 8-week postrandomization assessment, participants were presented with the statement “I found iWaWa useful” rated on a 5-point Likert scale (1=strongly disagree to 5=strongly agree).

##### Satisfaction

The Client Satisfaction Questionnaire (CSQ-8) [27,28] was used to assess treatment satisfaction. The CSQ-8 consists of 8 items rated on 5-point Likert scale. The CSQ-8 demonstrated excellent psychometric properties [37]. The CSQ-8 was adapted for this study by substituting “service” with “help” and “program” with “iWaWa.” The overall sum ranged from 8 to 32 with higher score indicating higher satisfaction.

##### Helpfulness

The 8-week postrandomization assessment also included 6 items developed for this study and designed to measure perceived helpfulness with anxieties and worries (eg, “Using iWaWa made it easier to cope with my worries”) on 5-point Likert scale (1=strongly disagree to 5=strongly agree).

#### Mental Health

Anxiety was measured using the GAD-7 [35]. The GAD-7 is a 7-item anxiety measure, and items are rated on 4-point Likert-scale ranging from 0 (not at all) to 3 (nearly every day). It demonstrated validity and reliability in clinical practice and research [35]. It has been suggested that the GAD-7 is a viable postpartum anxiety screening tool [38].

The Depression, Anxiety, and Stress Scale (DASS-21) [39] consists of 21 items rated on a 4-point Likert-scale ranging from 0 to 3. Higher scores indicate more severe symptoms. The DASS-21 has good internal consistency and concurrent validity [40,41].

#### Participant Characteristics

In line with the Consolidated Standards of Reporting Trials of Electronic and Mobile Health Applications and onLine TeleHealth (CONSORT-EHEALTH) checklist, demographics relevant to the digital divide as well as maternity characteristics were collected as part of the baseline questionnaire: age, ethnicity, education, employment, annual household income, relationship status, availability of computer, laptop, tablet, smartphone, number of previous children, and the number of weeks since giving birth.

#### Qualitative Treatment Feasibility and Acceptability Evaluation

##### Comments

Participants could write an optional comment about their experience at the end of each iWaWa module and in the follow-up assessments.

##### Follow-Up Interviews and Survey

All treatment group participants (including dropouts) were invited to take part in an optional semistructured phone interview or Web-based survey to collect in-depth information about their treatment experience. Participants gave verbal consent before the start of the interview. Interviews were audio-recorded and conducted by the first author (MA) using a semistructured interview schedule with open-ended questions (see Multimedia Appendix 2). The same questions were used for the Web-based survey for which consent had to be provided electronically. Participants received a £10 Amazon voucher as a compensation for their time.

### Data Analysis

#### Quantitative Data

Statistical analyses were performed with SPSS using a *P*<.05 significance level. Descriptive statistics including means, standard deviations, percentages, and proportions were used to describe the characteristics of the overall sample and the two groups, as well as the iWaWa program feasibility and acceptability and study feasibility. Independent *t* tests and chi-square tests were used to explore whether participant characteristics differed between the groups or between participants who did and did not complete the follow-up assessments.

For the mental health measures, group differences and differences over time were analyzed using independent and dependent sample *t* tests. Due to the large amount of missing data for the follow-up assessments, an intention-to-treat analysis was deemed inappropriate [42], and only the data of those completing the assessments were compared. Two-tailed bivariate correlations were conducted to explore whether there is a relationship between anxiety scores and the variables “weeks postpartum” and “number of children.”

#### Qualitative Data

The interview recordings were transcribed verbatim with all identifying information removed. Subsequently, the interview transcripts and the comments on the individual chapters were analyzed using inductive thematic analysis [43]. The software Quirkos was used to ensure systematic coding. The analysis identified general themes emerging form the comments and interviews.

## Results

### Participant Characteristics

Table 1 presents detailed information about participant characteristics of all randomized participants, those lost to follow-up, and those completing the 8-week follow-up assessment.

Participants were predominantly “Caucasian” (84/89, 94%), married (62/89, 69%), living with their partner/husband (79/89, 89%), and aged between 22 and 43 years (mean 32.02 years [SD 4.15 years]). Over half of the women had a bachelor’s degrees or higher and were on maternity leave. Slightly less than half of the women (38/89, 43%) had an income below £50,000 and about half (44/89, 49%) an income equal to or above £50,000. Women were between 1 and 52 weeks postpartum (mean 28.58 [SD 13.76]) and 72% (64/89) were first-time mothers (range 1-5 children [mean 1.36 (SD 0.68)]). The majority of participants reported having access to two or more technological devices (84/89, 94%). The two most commonly accessible devices were smartphone (88/89, 99%) and laptop (74/89, 83%).

**Table 1:** Participant characteristics of all randomized participants, those who were lost to follow-up, and those who completed the 8-week follow-up assessment.

Characteristic		All randomized participants			8-week follow-up assessment dropouts		8-week follow-up assessment completers	
		Total (n=89)	T^a^ (n=46)	WLC^b^ (n=43)	T (n=38)	WLC (n=22)	T (n=8)	WLC (n=21)
Age, mean (SD)		32.02 (4.15)	32.41 (3.55)	31.60 (4.71)	32.16 (3.80)	31.82 (5.30)	33.63 (1.69)	31.38 (4.13)
Number of children, mean (SD)		1.36 (0.68)	1.39 (0.77)	1.32 (0.57)	1.37 (0.79)	1.32 (0.58)	1.50 (0.76)	1.22 (0.58)
**Ethnicity, n (%)**								
	Caucasian	84 (94)	43 (93)	41 (95)	35 (92)	20 (91)	8 (100)	21 (100)
	Asian	4 (4)	2 (4)	2 (45)	2 (5)	2 (9)	0 (0)	0 (0)
	Mixed or multiple ethnicity	1 (1)	1 (2)	0 (0)	1 (3)	0 (0)	0 (0)	0 (0)
**Highest level of education, n (%)**								
	GCSE^c^	4 (4)	2 (4)	2 (5)	2 (5)	1 (4)	0 (0)	1 (5)
	A-level	18 (20)	7 (15)	11 (26)	7 (18)	7 (32)	0 (0)	4 (19)
	Bachelor’s degree	38 (43)	20 (44)	18 (42)	18 (47)	9 (41)	2 (25)	9 (43)
	Master’s degree	20 (23)	12 (26)	8 (19)	9 (24)	4 (18)	3 (37)	4 (19)
	Doctorate	4 (4)	3 (6)	1 (2)	1 (3)	0 (0)	2 (25)	1 (5)
	Other	5 (5)	2 (4)	3 (7)	1 (3)	1 (5)	1 (13)	2 (10)
**Current occupation, n (%)**								
	Student	2 (2)	0 (0)	2 (5)	0 (0)	1 (4)	0 (0)	1 (5)
	Employed (full-time,part-time, or self)	25 (28)	18 (39)	7 (16)	16 (42)	4 (18)	2 (25)	3 (14)
	Housekeeperor unemployed	10 (11)	3 (6)	7 (16)	2 (5)	5 (23)	1 (12)	2 (10)
	Maternity leave	48 (54)	23 (50)	25 (58)	18 (47)	11 (50)	5 (63)	14 (67)
	Other	4 (4)	2 (4)	2 (5)	2 (5)	1 (4)	0 (0)	1 (5)
**Household income, n (%)**								
	<£10,000-£19,999	7 (8)	2 (4)	5 (12)	2 (11)	3 (14)	0 (0)	2 (10)
	£20,000-£39,999	17 (19)	10 (22)	7 (16)	8 (21)	4 (18)	2 (25)	3 (14)
	£40,000-£59,999	24 (27)	14 (30)	10 (23)	12 (32)	4 (18)	2 (25)	6 (28)
	£60,000-£79,999	15 (17)	9 (20)	6 (14)	7 (18)	3 (14)	2 (25)	3 (14)
	≥£80,000	19 (21)	9 (20)	10 (23)	7 (18)	4 (18)	2 (25)	6 (29)
	Prefer not to say	7 (8)	2 (4)	5 (12)	2 (10)	4 (18)	0 (0)	1 (5)
**Relationship status, n (%)**								
	Singleor separated	6 (7)	2 (4)	4 (9)	2 (5)	2 (9)	0 (0)	2 (9)
	Married or in a relationship	82 (92)	44 (96)	38 (88)	36 (95)	19 (86)	8 (100)	19 (90)
	Prefer not to say	1 (1)	0 (0)	1 (2)	0 (0)	1 (4)	0 (0)	0 (0)
Weeks postpartum, mean (SD)		28.58 (14)	29.70 (14)	27.40 (14)	30.97 (14)	26.50 (15.)	23.63 (14)	28.33 (12)

^a^T: treatment group.

^b^WLC: wait-list control group.

^d^GCSE: general certificate of secondary education.

**Figure 2:**
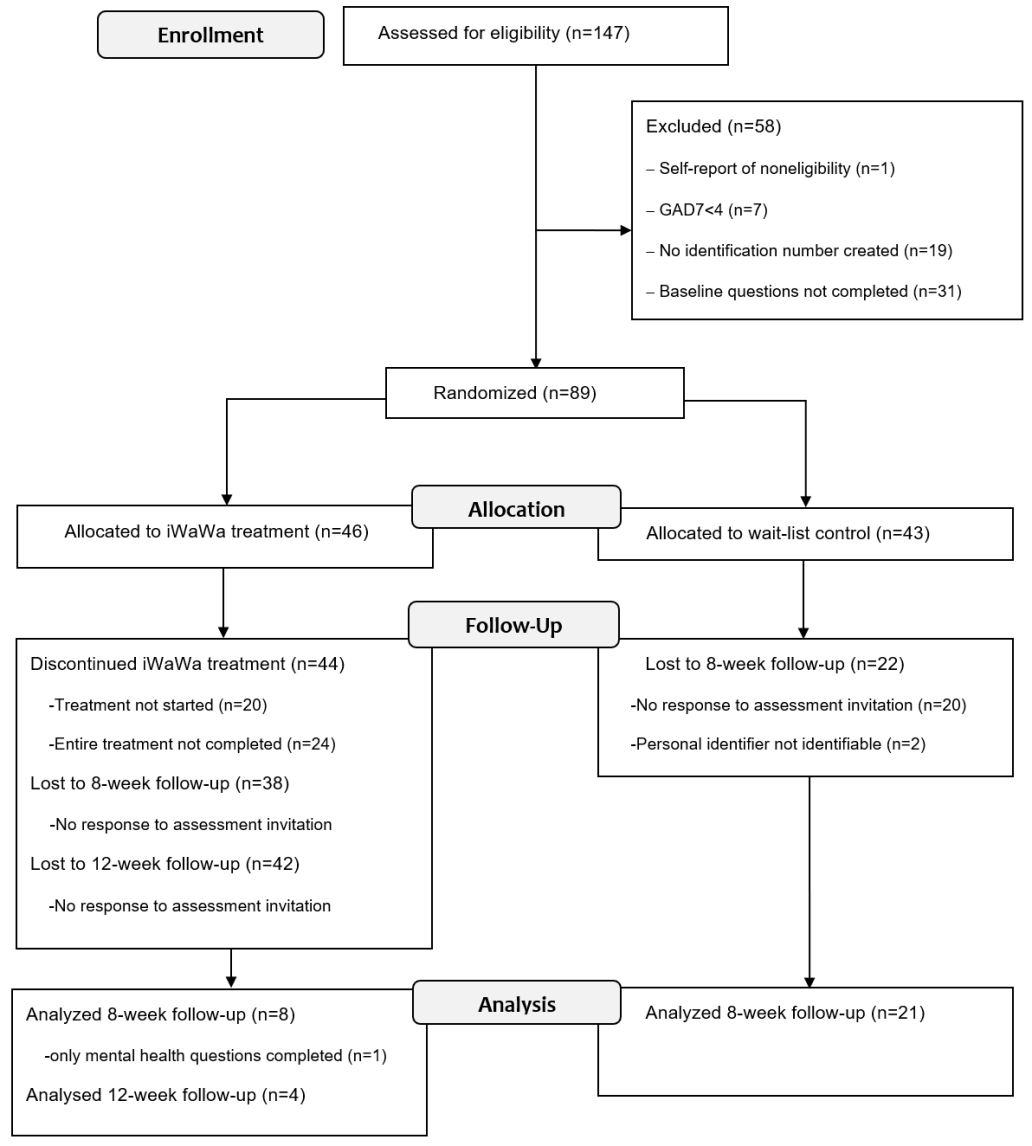
Flowchart of participants flow through all trial stages. iWaWa: Internet-based What Am I Worried About; GAD7: Generalized Anxiety Disorder Scale.

There were no significant differences between the treatment and wait-list control groups in demographic characteristics, except for relationship status (*P*=.03). No differences were found between participants of the treatment and wait-list group who completed the 8-week follow-up assessment, and no differences were found between participants of the treatment and wait-list group who did not complete the 8-week follow-up assessment. There were also no differences between treatment group participants who started the iWaWa treatment and those who did not.

### Study Feasibility

#### Recruitment

Figure 2 presents the CONSORT diagram showing participant flow and attrition through the trial. During the recruitment, 147 women accessed the initial assessment and consented to take part. A total of 58 (39%) were excluded (see Figure 2 for exclusion reasons). The remaining 89 (60%) were randomized into the treatment (n=46) or wait-list control groups (n=43).

Of the 89 randomized women, 86 (97%) had heard about the study from Facebook and 3 (3%) from friends.

#### Dropout Attrition

A total 21 out of 43 wait-list control participants (48.84%) completed the 8-week follow-up assessment. Eight of 46 (17%) treatment group participants responded to the 8-week follow-up assessment (one participant completed only the GAD-7 and DASS-21). There was a significant difference in attrition rates between the groups (*P*=.002). Four of 46 (9%) women in the treatment group completed the final (12-week follow-up) assessment. One of the four had not completed the 8-week follow-up assessment.

### Treatment Feasibility

#### Engagement

##### iWaWa Modules

**Figure 3:**
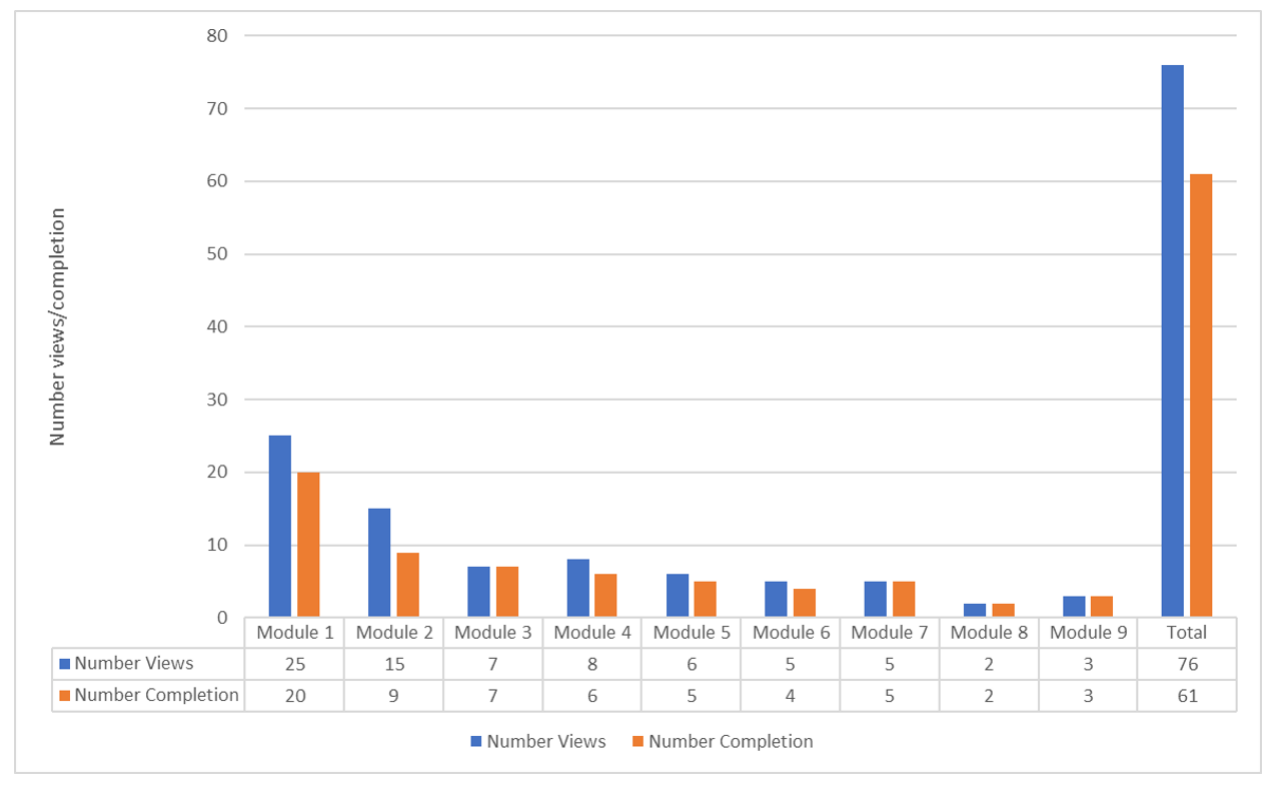
Treatment group Internet-based What Am I Worried About (iWaWa) module views and completion.

Of the 46 treatment group participants, 26 participants (56%) viewed at least one module and on average 1.65 modules (SD 2.51; including repeat views). Two participants viewed all modules (4.34%). Figure 3 illustrates the number of module views and completion by the treatment group. Of the 76 modules viewed, 61 (80.26%) were completed. Module 1 was most viewed with a marked reduction thereafter. Engagement with the 14 interactive components within the program ranged from 50% to 100% (mean 69 [SD 0.17]).

##### Email, Text, and Phone Support

Of the 25 women in the treatment group accessing module 1, 24 (96%) signed up for weekly reminders (email: n=16, text: n=6, email and text: n=2). One woman requested a support call but did not respond when asked about a call date and time.

#### Usability

The average SUS usability score was 40.00 (SD 15.76; n=9), indicating a usability below average. Module 2 was experienced as least clear and understandable (mean 4.25 [SD 1.75]), and module 1 as most clear and understandable (mean 6.20 [SD 0.62]). Out of 8 respondents, 5 (62%) did not agree with the statement that they could use iWaWa seamlessly as part of their daily routine. With the statement regarding regular usage after the study ended, 2 participants disagreed (25%). Regarding technical issues, iWaWa had a 2-day downtime due to a broken link.

### Treatment Acceptability

#### Usefulness and Satisfaction

Module 8 was experienced as least useful (mean 3.50 [SD 2.12]) and module 7 as most useful (mean 6.00 [SD 0.82]). Of the 8-week postrandomization assessment respondents, 71% (5/7) rated iWaWa as useful, 14% (1/7) rated iWaWa as neither useful nor not useful, and 14% (1/7) as not useful. The average treatment satisfaction CSQ-8 score was 20.22 (SD 5.61) on a range of 8-31.

#### Helpfulness

Of the 7 respondents, 71% (5/7) agreed that iWaWa helped them better understand anxiety, 57% (4/7) agreed that it helped them develop skills to manage anxiety, 57% (4/7) agreed that it helped them manage their unhelpful thoughts, 42.86% (3/7) agreed that it helped reducing distressing bodily sensations, 28% (2/7) agreed that it improved their well-being, and 43% (3/7) agreed that it made it easier to cope with their worries.

### Qualitative Treatment Feasibility and Acceptability Outcomes

Qualitative data comprised 31 comments from iWaWa modules and follow-up questionnaires and five interviews (13-18 min). Data saturation was assumed when no new themes emerged from the interviews and comments. Of the interviewees, one completed the first module and the remaining four between 4 to 9 modules.

#### Themes

**Figure 4:**
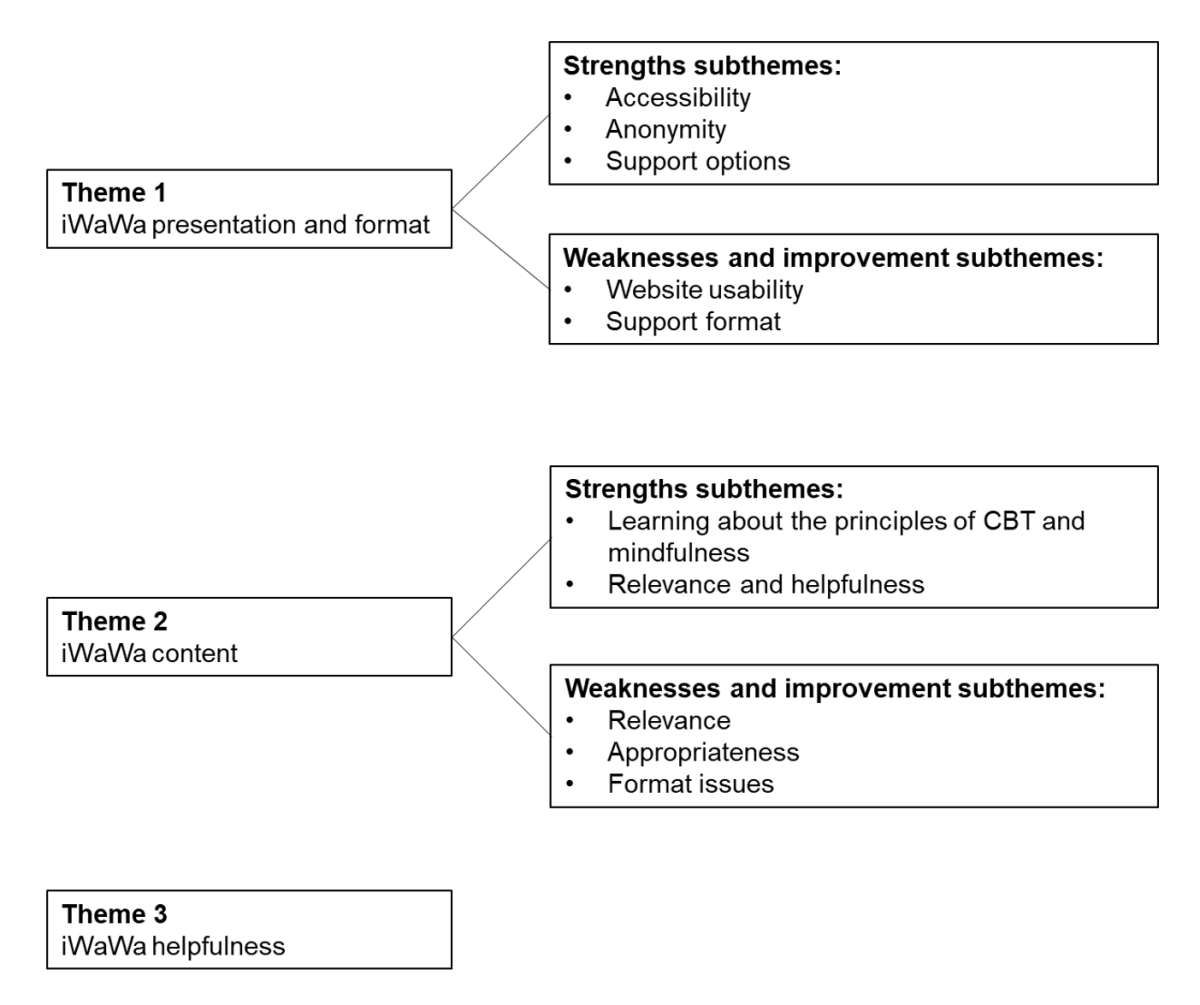
Diagram of qualitative themes and subthemes. CBT: cognitive-behavioral therapy; iWaWa: Internet-based What Am I Worried About.

Thematic analysis generated 3 key themes (presentation and format, content, and helpfulness) and 10 subthemes. Figure 4 presents a diagram of the themes and associated subthemes. Textbox 1 contains quotes for each theme and subtheme.

Overall, participants described iWaWa as generally useful and straightforward but not user-friendly. Participants reported feeling that it was good to know that something like iWaWa is being developed and that it should be further developed to make it accessible to more women in the same situation.

##### Theme 1: iWaWa Presentation and Format

###### Strengths Subthemes

####### Accessibility

Participants enjoyed that iWaWa could be accessed from home at a time that suited them, which was reported important with a newborn baby and multiple children.

####### Anonymity

Women appreciated iWaWa’s anonymity. One participant stated that she would have not reached out for help any other way.

####### Support Option

The weekly email and text messaging reminder option was described as a strength of iWaWa and the support phone calls as a valuable option.

###### Weaknesses and Improvement Subthemes

####### Website Usability

It was reported that iWaWa did not display well on their smartphones and that iWaWa was not very user-friendly and modern. Frustration was caused by having to find the iWaWa link for logging in. All stated they would prefer iWaWa as an easy to use smartphone app.

**Quotes for all themes and subthemes of the thematic analysis.**

Theme 1

- Strengths subthemes: Accessibility
  - Interview 1: *I like the fact that I could do it in my own time at home...cause I have three children so it wasn't like I would have to try to have to make appointments and get child care so I could do it when they were in bed or you know whenever it sort of seemed to fit in with my lifestyle I suppose.*
  - Interview 3: *I found it quite easy especially while I was breastfeeding on my phone or my tablet, so that was good.*

- Strengths subthemes: Anonymity
  - Interview 5: *I generally feel pretty confident and happy and would not label myself as somebody with anxiety or any of those issues so I don't think I would have accessed help in another way because I didn't really want to be labeled as you know as an anxious or depressed or whatever.*

- Strengths subthemes: Support options
  - Interview 3: *The reminders were good cause I would have forgotten about it otherwise, you know to do it. It was nice to have the link on the email rather than having to find the original one.*
  - Interview 2: *I suppose for some people some of the modules might sort of trigger feelings or bring up things that were a bit difficult for them so I think having that option of support [call] is definitely useful.*

- Weaknesses and improvement subthemes: Website usability
  - Module 1, Comment 3: *The pages weren't phone friendly- lots of scrolling left to right.*
  - Module 1, Comment 4: *Doesn't work that well on an iPhone. Few mums have time to sit at the computer.*
  - Interview 5: *I think the sort of user experience and interface and how you accessed it felt very old-fashioned compared to you know apps that feel a lot more kind of modern and easy to access on mobile and therefore fitting with your life a lot more easily.*
  - Interview 4: *You have to keep clicking next and go to the next page...because if you're internet connect is not great and you click next then it takes a while for the next page to come up and you know it gets frustrating.*
  - Interview 2: *I don't use apps very often but yeah if that could solve the presentation issue it I might have continued a bit longer.*

- Weaknesses and improvement sub-themes: Support format
  - Interview 2: *I find it really hard to have any kind of phone conversation most of the time because you are always sort of having to jump up and hold the baby and deal with crying and even a 5-minute phone call could be quite challenging…I kind of just didn't have the energy and the time to engage in that.*
  - Interview 5: *I didn't feel like I needed the other support...and I suppose it wasn't clear to me who the coach was and what their qualification or skills were and if it was help you with technical issues or to help you with or offer you more support with the with your worries.*

Theme 2

- Strengths subthemes: Learning about the principles of CBT and mindfulness
  - Interview 5: *I think that the whole of the first section explaining the principles themselves just absolutely completely changed the way I thought about certain things and helped me to go and put other things place in my life that helped me managed the anxiety so I went and signed up for a mindfulness app...and the fact that reframing unhelpful thoughts I find all of the principles really really useful and have applied it in lots of ways so that was the biggest thing for me with actually seeing ways that I could manage these sort of out of control worries without you know I don't know spending hours on the Internet.*

- Strengths subthemes: Topic relevance and helpfulness
  - Interview 2: *I think cause you forget very quickly so it was pretty much just as relevant if it would have been the first time and you know each baby is different.*
  - Interview 4: *It makes you realize that there are obviously other women who are experiencing these similar things and the fact that it's being written down by a professional some of those things and anxieties that I have they haven't listened to me and they have already written that and it makes you realize that there are other people with the same worries and even that can help make you feel better.*

- Weaknesses and improvement subthemes: Relevance
  - Interview 5: *I didn't think about when your baby cries and how it makes you feel which I never had a concern or a problem with that so that just to me was too specific to make it feel like it was worth my time going through it.*
  - Module 3, Comment 2: *We didn’t have any problems with breastfeeding. Also my daughter is now 5 months so a bit too late for us.*

- Weaknesses and improvement subthemes: Appropriateness
  - Module 2, Comment 1: *The helpful actions were a bit ridiculous. Putting a baby down won’t stop them crying. How can I have a cup of tea if they're still crying. The useful actions need to be rephrased about calming yourself down or give tips on how to calm a baby.*
  - Interview 1: *It came across sometimes as if it was approving of a certain type of parenting if that makes sense… So like with my first I ended up with a baby that would wake up every time that I would put her down and she would only sleep if I carried her around in the sling or she was in bed with me and it felt sometimes and that gave me a lot of anxiety because everyone around me was sort of saying you're gonna make a rod for your own back and I think if I had come across this program at that point I think the way some of it was written would have given me more anxiety… I sort of felt as if that would might be pushing some mums to make a decision that didn't feel right to them because they felt like that's what they should be doing sort of thing.*
  - Module 9, Comment1: *This course has been a great resource but I really feel it needs to be more inclusive of ALL parenting approaches in order not to increase the anxiety of those who have either chosen or fallen into a different path because that’s what works for them. Statement such as “I can explore different settling methods to see what works best for my baby” would be far better.*

- Weaknesses and improvement subthemes: Format issues
  - Interview 4: *In the evening once my baby was asleep and by the time I've eaten and done chores you're really tired and I found it just sometimes quite difficult cause there were yeah so many words.*
  - Interview 2: *I found it really hard to generate my own response, just when you are really tired and you haven't got much time, you got a crying baby...I suppose you could have a drop-down menu where you could choose from a list and then you could have space where could write in if they want.*

Theme 3

- iWaWa helpfulness
  - Interview 1: *I still use them [mindfulness exercises] now if am feeling anxious then I try and concentrate on the moment and remember my breathing and all that so yeah I did definitely find it useful for me.*
  - Interview 2: *I think maybe to a fraction...I think it's got a lot of potential if it was designed in a really user-friendly quick easy way it could be a lot more useful you know the principle of it is good just a little bit of hard work.*

####### Support Format

Regarding the support calls, women mentioned the importance of further highlighting the support call option and its purpose and credibility and more frequent reminders. One participant would have preferred a more anonymous option (eg, email or text chat).

##### Theme 2: iWaWa Content

###### Strengths Subthemes

####### Learning About the Principles of Cognitive-Behavioral Therapy and Mindfulness

Women stated to have especially enjoyed the first module and felt the module provided them with “tools” that some were still using, for example, by downloading mindfulness apps.

####### Relevance and Helpfulness

Women stated that most included topics (anxieties) were generally relevant. Women described it as helpful to learn that other women experience same or similar anxieties.

###### Weaknesses and Improvement Subthemes

####### Relevance

Many participants stated that not all topics were as relevant and that some were too specific, which made them skip modules. Women with multiple children felt that iWaWa could benefit from making the content more applicable to their situation.

####### Appropriateness

Several women reported to have experienced the content, especially the unhelpful and helpful actions and examples, as “ridiculous” and “a bit off-putting” and promoting a certain parenting style. It was suggested that the program content should be reflecting a range of parenting styles.

####### Format Issues

Participants felt that some of the content was repetitive and very “wordy” and could be improved by having more concise and shorter modules. It was mentioned that it was “labor-intensive” to generate their own examples for the exercises and that the exercise examples were described as “hard to relate to.” It was suggested to offer the option of using “pre-provided” statements for the exercises.

##### Theme 3: iWaWa Helpfulness

Of the 5 interviewed women, 2 felt that iWaWa helped with their anxieties, 2 felt that it helped a bit, and 1 said that it probably would have helped if she had done more of the program. All felt that iWaWa could be more helpful with their anxieties if the presentation and content was improved.

**Table 2:** Mental health levels and scores at baseline, 8-week follow-up, and 12-week follow-up.

Measure		Baseline, mean (SD)			8-week follow-up, mean (SD)			12-week follow-up, mean (SD)
		Total (n=89)	T^a^ (n=46)	WLC^b^ (n=43)	Total (n=29)	T (n=8)	WLC (n=21)	T (n=4)
GAD-7^c^		12.26 (4.21)	12.46 (3.96)	12.05 (1.98)	7.83 (4.52)	6.63 (5.29)	8.29 (4.24)	8.75 (4.03)
**DASS-21^d^**								
	Depression	8.16 (4.47)	8.28 (3.91)	8.02 (5.05)	4.34 (3.83)	2.75 (3.33)	4.95 (3.91)	4.75 (3.30)
	Anxiety	6.52 (3.81)	6.22 (3.41)	6.84 (4.21)	3.90 (4.06)	3.38 (1.85)	4.10 (4.66)	3.00 (2.16)
	Stress	11.82 (3.80)	11.63 (3.59)	12.02 (4.04)	8.38 (3.71)	7.13 (3.14)	8.86 (3.86)	10.75 (3.40)

^a^T: treatment group.

^b^WLC: wait-list control group.

^c^GAD-7: Generalized Anxiety Disorder Scale.

^d^DASS-21: Depression, Anxiety, and Stress Scale.

### Mental Health Outcomes

Table 2 contains the mental health outcomes for the treatment and wait-list control groups at baseline, the 8-week follow-up, and 12-week follow-up. A total of 28% (25/89) women scored in the mild anxiety range, 42% (37/89) in the moderate anxiety range, and 33% (27/89) in the severe anxiety range on the GAD-7 at the baseline assessment. There were no significant differences between the treatment and wait-list control groups on all mental health measures at baseline. At the 8-week assessment, no significant group differences were found for anxiety (GAD-7 and DASS-21 anxiety). For both groups, anxiety scores significantly reduced from baseline to the 8-week follow-up. There was no significant difference between the 8-week and 12-week follow-up in anxiety scores for the treatment group. Multimedia Appendix 3 illustrates the GAD-7 and DASS-21 anxiety scores for the 3 participants who started the iWaWa program and completed both follow-up assessments. There was no significant correlation between any of the anxiety scores and time since birth and number of children.

## Discussion

### Principal Findings

This study aimed to assess the trial’s feasibility, iWaWa’s treatment feasibility and acceptability, and explore changes in postpartum anxiety in association with iWaWa. Regarding the study’s feasibility, the minimum sample size required was exceeded within the relatively short recruitment period (8 weeks). Facebook proved most successful for recruitment. However, there was a high dropout attrition from the study and also a high treatment nonusage attrition. Among women who accessed iWaWa, the treatment was experienced as generally useful and helpful but not user-friendly enough in terms of treatment format and content. Participants felt that iWaWa could be improved by having it as a smartphone app and by making the content more concise and inclusive of different parenting styles. Anxiety levels decreased significantly for both groups from baseline to the 8-week follow-up assessment, but there were no differences between the treatment and wait-list control group. However, due to high drop-out and nonusage attrition, especially in the treatment group, the results for mental health cannot be interpreted reliably and will therefore not be further discussed. The results regarding recruitment and attrition, usability, and acceptability will be discussed in more detail below, including a comparison with previous literature and potential program improvements.

### Recruitment and Attrition

Within a relatively short recruitment period, the iWaWa trial successfully recruited more than the minimum calculated sample size through Facebook. The response rate is comparable to similar studies. A study recruiting women with postpartum anxiety for a Web-based online survey through Facebook had a similar response rate (220 respondents over 4 months) [44]. Two studies recruiting women in the United Kingdom with postpartum depression through website advertisement banners for a Web-based treatment had similar (249 respondents over 5 months [33]) and higher response rates (1403 respondents over two waves of 2-week recruitment periods [34]). The successful recruitment might indicate that there is an interest and potential need for a treatment such as iWaWa among postpartum Facebook users. Due to the inability to recruit participants in health care settings, it remains to be investigated whether recommendation or endorsement from health care professionals might have increased iWaWa’s recruitment rates.

Dropout attrition in the iWaWa trial was high (49% to 91%). In comparison, in the open pilot study evaluating WaWa, dropout attrition ranged from 39% to 61%. However, it has been pointed out that many trials testing Web-based interventions often suffer from high attrition rates [45]. Due to the lack of other trials of Web-based postpartum anxiety treatments, attrition can only be compared with trials of postpartum depression. Regarding postpartum Web-based treatments, one study evaluating the feasibility for depression with minimal support (Netmums program) also reported a high attrition rate (76%) [34]. The study found that, like this trial, there was an initial high access with a small user subgroup continuing to access and use the program. The authors discussed the role of curiosity, and it has also been suggested that one session may satisfy the user’s need [46]. The Netmums program’s attrition reduced to approximately 20% at the posttreatment assessment when telephone support was added [33]. Similarly, the Web-based MomMoodBooster program for postpartum depression with telephone guidance also reported comparably very low attrition rates [31,46]. When comparing iWaWa’s attrition to postpartum depression trials, it has to be noted that a systematic review found higher Web-based intervention adherence rates for depression than anxiety [47].

There are a variety of factors that could have caused iWaWa’s high attrition. Commonly identified reasons for dropout include the burden of the program, program information structure, content relevance, level of support, technical access issues, time constraints, lack of motivation, and improvement of the group [34,47,48]. The potential role of these factors regarding iWaWa’s high attrition will be briefly discussed.

This study suggests that some women indeed experienced the access, length, and exercises of iWaWa as a burden. With the infant’s demanding schedule, it is possible that iWaWa participants felt a lack of motivation or experienced time constraints. For Web-based postpartum depression treatments, adherence was high when scheduled support was offered [31,33,46]. Scheduled support was part of the original WaWa treatment and valued as strength of the treatment [4]. On the basis of a survey which found that telephone support was not the preferred mean of support among women with postpartum anxiety [44], it was decided for iWaWa’s telephone support to be optional. Scheduled support might improve adherence of women wanting this type of support, but it might also put off women who do not feel the need or find it unsuitable. Offering to opt in for scheduled support at the treatment start might be an alternative for future iWaWa versions. Regarding content relevance, a qualitative study suggested that being able to identify with a an online intervention program helps with adherence in Web-based psychological interventions [48]. As some of iWaWa’s topics were not experienced as relevant, it could therefore be that some participants logged into iWaWa but dropped out because they experienced the topics as irrelevant. Concerning symptom improvement, a recent systematic review and meta-analysis found that the prevalence of postpartum anxiety decreases from 1-4 weeks to 5-12 weeks [1], and it could therefore be that the participant’s symptoms improved and they no longer felt the need for iWaWa. In addition, almost half of the participants scored in the severe to extremely severe range of stress. It might be that participants were in need for stress management techniques, which could be incorporated in future iWaWa versions. The iWaWa program used an organic information structure design that allowed users to freely explore the modules. The use of a tunnel design, in which users navigate through content in a sequential order, has been identified as less likely to overwhelm users with options [49] and may increase use [50].

### iWaWa’s Usability and Acceptability

Participants enjoyed iWaWa’s accessibility, anonymity, and weekly reminders, as well as the introduction to the principles of CBT and mindfulness. This is in line with previous qualitative research regarding Web-based treatments for postpartum depression [23,32,51], as well as postpartum UK health care professionals (health visitors) [52].

iWaWa was rated and experienced as generally useful and helpful but not user-friendly enough. The iWaWa content was experienced as too long. A preference for brief modules was also identified by Web-basedsurveys among a sample of adults [53] and among women with postpartum anxiety [44]. Participants also felt that iWaWa’s content was not inclusive of different parenting styles. WaWa’s content was found to be acceptable in a small sample (n=7, in the posttreatment evaluation interview) pilot study in Australia [4]. It might therefore be important to investigate in more depth the needs of a larger and more diverse sample of women with postpartum anxiety so that iWaWa’s content and format can be adapted to better meet their needs.

Women in this study also felt that not all topics were relevant. The need for Web-based treatments to be relevant to their own needs and circumstances was also discovered among women with postpartum depression [23,32]. Future iWaWa versions might benefit from presenting users with content most relevant to them (eg, anxiety topic needs and relevance assessment before the treatment starts).

Interactive components with space for responses were previously found to be valued among women with postpartum depression [23]. Even though most iWaWa users engaged with the exercises, many experienced them as difficult. The ease of usage of the interactive components seems important among iWaWa users, and therefore exercises of future iWaWa versions should be tested by potential users before implementation (eg, think-out aloud technique).

Regarding the format, iWaWa participants experienced the log-in process as difficult. The log-in process was also identified as difficult by women testing a Web-based postpartum depression treatment [32]. Participants also expressed that iWaWa was not smartphone-friendly, which was also reported by women testing a Web-based treatment for postpartum depression [32]. The preference for a smartphone-compatible treatment was also found in a survey among women with postpartum anxiety [44]. iWaWa was smartphone compatible, but iWaWa as a smartphone app might improve the ease of access and presentation on smartphones.

### Limitations

The generalizability of the results of this study is compromised due the use of convenience sampling and a homogenous sample. Findings may therefore not be representative of women from different cultural or ethnic backgrounds, lower socioeconomic status, and more severe anxiety. In addition, most women were recruited through Facebook, so findings are limited to this self-selected pool of women. However, the homogeneous nature of this sample can also be a strength, as results can be generalized for this specific group. Furthermore, no incentives were offered during the enrollment, allowing for a sample with a genuine and intrinsic need. The generalizability may also be affected by the fact that the anxiety status was established by a screening instrument and not by a diagnostic tool. However, the GAD-7 is a frequently used anxiety screening tool and has been suggested as suitable for postpartum anxiety [38]. In addition, diagnostic interviews are less anonymous and iWaWa’s anonymity was highlighted as an important strength by the participants.

High dropout and nonusage attrition may have also biased the results. Therefore, findings regarding iWaWa’s usability, acceptability, and mental health changes are limited to the experiences and views of a small sample. None of the women who did not start iWaWa took part in the follow-up interviews; so, no knowledge could be gained regarding what caused the early dropout.

The interviewer of this study was MA, who is a doctoral student investigating Web-based treatments for postpartum anxiety with personal experience related to this subject. These experiences may affect the collection, analysis, and interpretation of the data. Therefore, several actions were taken to minimize the likelihood of this risk. Participants were aware that the interviewer was the main study lead, but reassured of the importance of any feedback (positive and negative) for improving iWaWa. The qualitative analysis also included comments from the iWaWa modules and the follow-up assessment in which women might have felt more comfortable providing negative feedback. MA kept a reflective journal to consider how the treatment and interviewee responses affected her own views on iWaWa throughout data collection, analysis, and reporting. An effort was made to consider this when analyzing the data. Interviewees were offered monetary compensation for their time, which might have confounded their responses.

### Conclusions

This study demonstrated that there is an interest in a postpartum anxiety Web-based treatment like iWaWa. However, the iWaWa study and program in the current format is not yet feasible and acceptable, and due to high dropout rates, the results on the impact of iWaWa on anxiety cannot be interpreted reliably. Nonetheless, the study revealed strengths and weaknesses of the iWaWa content and format, as well as highlighted potential areas of improvements. As a first study investigating the usability and acceptability of Web-based treatment specifically targeted at postpartum anxiety, these results provide useful information about Web-based treatment preferences that can help improve iWaWa and inform research and development to optimize usability, acceptability, and Web-based treatment adherence in this population. This study also contributes to filling the gap in evidence-based self-help for mild-to-moderate postpartum anxiety symptoms.

## Appendix

Multimedia Appendix 1 Two images of the iWaWa (Internet-based What Am I Worried About) program.

Multimedia Appendix 2 Follow-up interview schedule.

Multimedia Appendix 3 Illustration of the anxiety scores of study completers and iWaWa (Internet-based What Am I Worried About) starters for all assessments.

Multimedia Appendix 4 CONSORT‐EHEALTH checklist (V 1.6.1).

## Abbreviations

CBT: cognitive-behavioral therapy
CONSORT-EHEALTH: Consolidated Standards of Reporting Trials of Electronic and Mobile HEalth Applications and onLine TeleHealth
CSQ-8: Client Satisfaction Questionnaire
GAD-7: Generalized Anxiety Disorder Scale
iWaWa: Internet-based What Am I Worried About
RCT: randomized controlled trial
SUS: System Usability Scale
WaWa: What Am I Worried About
