# Supplementary material for: Feasibility and Acceptability of a Web-Based Treatment with Telephone Support for Postpartum Women With Anxiety: Randomized Controlled Trial
Source: JMIR Ment Health. 2018 Apr 20;5(2):e19. doi: 10.2196/mental.9106 (PMC5938691; doi:10.2196/mental.9106)
Supplement: Multimedia Appendix 1 [file mental_v5i2e19_app1.pdf]

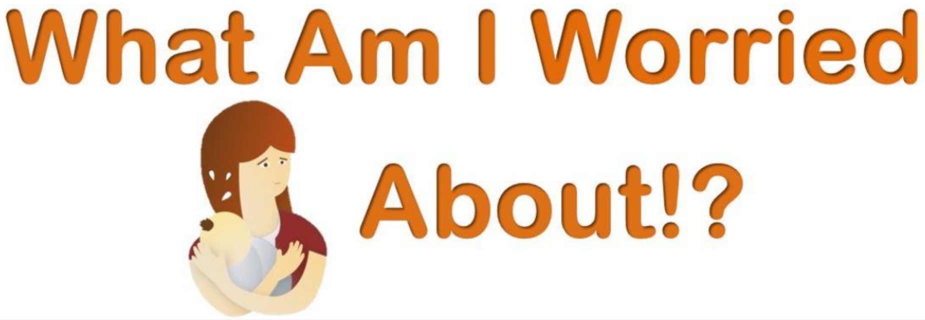

Welcome to the iWaWa program.

The program consists of **nine chapters** which you are asked to go through in the following 8 weeks.

Below you can see **our recommended order** of the chapters organised per week. You can **pick any order** for the Practice chapters, but it is important to **start with Chapter 1 “Is this for me?”** and **end with Chapter 9 “Understanding”** and go through all nine chapters by the end of the following 8 weeks.

You can go through a chapter in one go or you can come back and continue where you left off (make sure to use the same browser if you want to get back to where you left off). You can also access the chapter as often as you want to. If you have any questions or issues please contact Miriam T. Ashford (Miriam.Ashford@city.ac.uk).

To get started, click on the “Is this for me?” chapter below.

Week 1

Chapter 1: Is this for me?

Week 2 – Week 7: Practice chapters

Chapter 2: When My Baby Cries

Chapter 3: Feeding My Baby

Chapter 4: Managing Inconsistent Information

Chapter 5: My Baby’s Health And Safety

Chapter 6: Leaving My Baby To Be Cared For By Someone Else

Chapter 7: Comparing Myself With Others Or The Way I Used To Be

Chapter 8: Leaving the House To Go Out

Week 8

Chapter 9: Understanding

Thank you very much for using the iWaWa program and participating in our study!

What Am I Worried About!?

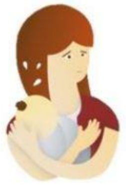

What Is This Program About?

All mothers want to feel calm and confident. **Feeling worried is normal while adjusting to new life experiences such as caring for a baby**.

Do you sometimes **feel overwhelmed and agitated**? Are your **worries, doubts and fears interfering with your daily life** and **preventing you from experiencing times of enjoyment with your baby and your family?**

These feelings can be quite normal but, if you feel like this every day, most of the time, or if your worries are very intense, then anxiety could be reducing your quality of life as a mother.

There are things that everyone can do to help themselves feel better and enjoy this phase of life more.

This online self-help program is a first step towards easing distress and feeling more confident and calm.

This program is based on established theory. The practices are known to be effective in reducing anxiety. The ideas are designed to fit into the busy lives of mothers of young babies.

Previous Next

0% 100%

CITY UNIVERSITY OF LONDON EST 1894 MONASH University

Survey Powered By Qualtrics
