# Supplementary material for: Feasibility and Acceptability of a Web-Based Treatment with Telephone Support for Postpartum Women With Anxiety: Randomized Controlled Trial
Source: JMIR Ment Health. 2018 Apr 20;5(2):e19. doi: 10.2196/mental.9106 (PMC5938691; doi:10.2196/mental.9106)
Supplement: Multimedia Appendix 2 [file mental_v5i2e19_app2.pdf]

Follow-up Interview Schedule

A. Overall experience

1. Why did you decided to take part in this study? Was there anything that appealed to you?
2. Were you able to start using iWaWa? Is there anything that could have made it easier for you to start iWaWa? - **for non-starters only**
3. Were you able to read through all chapters? If not, what made you stop? Is there anything that could have made it easier for you to continue using iWaWa?

B. Acceptability & Usability

1. Were the iWaWa chapters/topics relevant to you? Were any topics missing?
2. What did you find most helpful with iWaWa?
3. What made it difficult for you to use iWaWa? How could we have made it easier?
4. How did you find iWaWa being delivered via the internet?
5. What do you think about the support available for iWaWa?
    i. In terms of weekly email/SMS reminders
    ii. In terms of iWaWa coach calls?

C. Other

1. Do you think iWaWa helped you with your anxiety? If yes, how? If no, why?
2. In your view, how could iWaWa be improved?
3. Do you have any other comments about what we have talked about today?
