# Supplementary figures and images for: Feasibility and Acceptability of a Web-Based Treatment with Telephone Support for Postpartum Women With Anxiety: Randomized Controlled Trial
Source: JMIR Ment Health. 2018 Apr 20;5(2):e19. doi: 10.2196/mental.9106 (PMC5938691; doi:10.2196/mental.9106)

Participant 1 (1 chapter viewed)

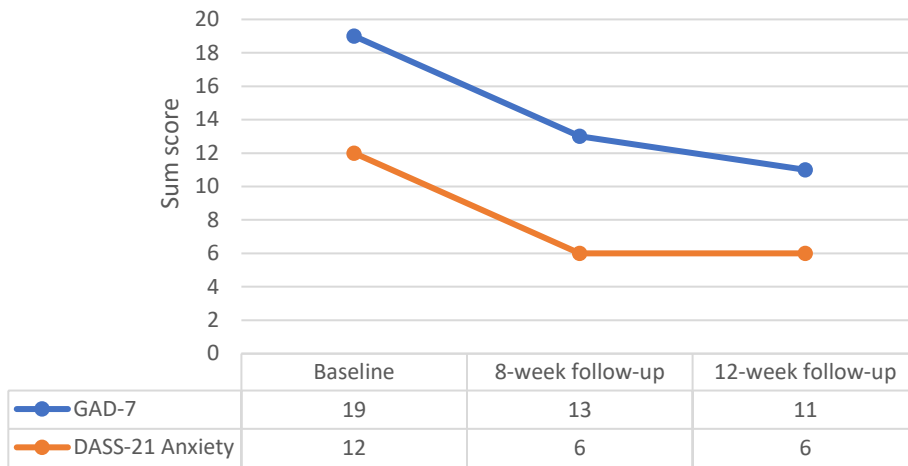

Participant 2 (4 chapters viewed)

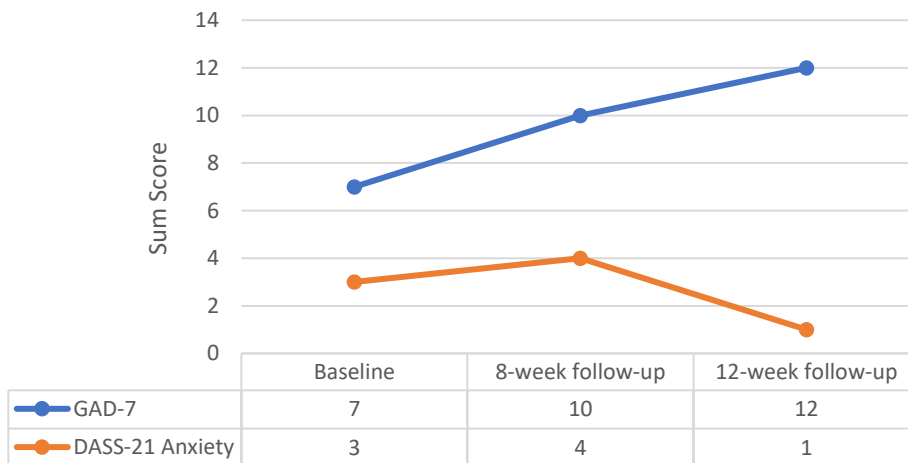

Participant 3 (9 chapters viewed)

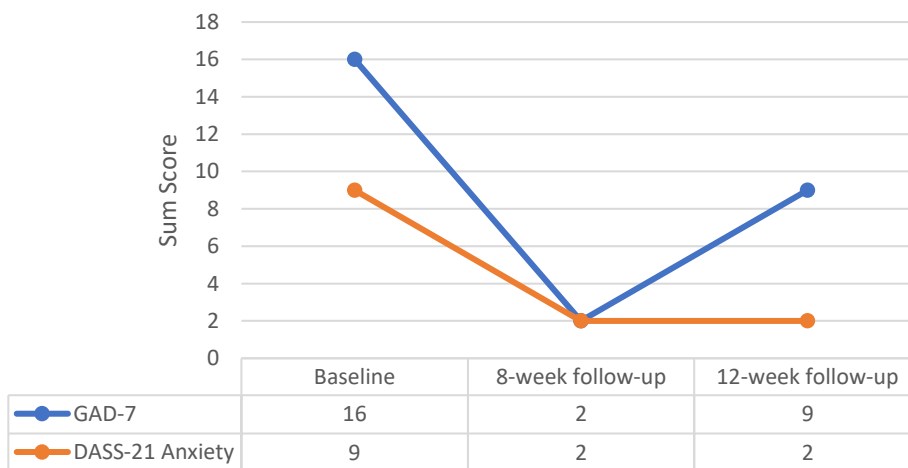

Supplement: Multimedia Appendix 3 [file mental_v5i2e19_app3.pdf]
